# Supplementary material for: Causal Relationship between Aging and Anorexia Nervosa: A White-Matter-Microstructure-Mediated Mendelian Randomization Analysis
Source: Biomedicines. 2024 Aug 16;12(8):1874. doi: 10.3390/biomedicines12081874 (PMC11351342; doi:10.3390/biomedicines12081874)
Supplement: Supplementary file 1 [file biomedicines-12-01874-s001.zip › Supplementary Figure/Figure S8/Leaveoneout plot of MR analysis for TL to ubm-b-2000.pdf]

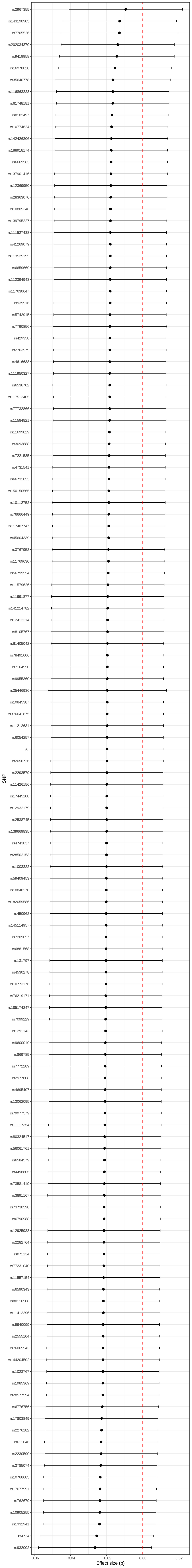
rs2967355
rs143190905
rs7705526
rs202034370
rs9419958
rs16978028
rs35640778
rs116863223
rs61748181
rs8102497
rs10774624
rs142426306
rs188918174
rs6669563
rs137901416
rs12369950
rs28363070
rs10805346
rs139795227
rs111527438
rs41269079
rs113525195
rs6659669
rs112394943
rs117630647
rs939916
rs5742915
rs7790856
rs429358
rs2763979
rs4616688
rs111950327
rs6536702
rs117512405
rs77732866
rs11584821
rs11699829
rs3093888
rs7221585
rs4731541
rs66731853
rs150150565
rs10112752
rs76666449
rs117407747
rs45604339
rs3767952
rs11769630
rs56799554
rs11579626
rs11991877
rs141214782
rs12412214
rs8105767
rs61405042
rs78491606
rs7164950
rs9955360
rs35446936
rs10845387
rs376641875
rs11212631
rs6054257
A8
rs2056726
rs2293579
rs11426156
rs17445108
rs12932179
rs2538745
rs139669835
rs4743037
rs28502153
rs1003322
rs59409453
rs10840270
rs182059586
rs450962
rs145114957
rs7209057
rs6881568
rs131797
rs4530278
rs10773176
rs76219171
rs185174247
rs7099229
rs1291143
rs9600019
rs869785
rs7772289
rs2977608
rs4695407
rs13062095
rs79977579
rs11117354
rs80324517
rs56061761
rs6584579
rs4498805
rs73581419
rs3891167
rs73730598
rs6790988
rs12925933
rs2282764
rs871134
rs77231040
rs11557154
rs6590343
rs80116508
rs11412296
rs9940099
rs2555104
rs76065543
rs144204502
rs1023767
rs1985369
rs28577594
rs6776756
rs17803849
rs2276182
rs611646
rs2230590
rs3785074
rs10768683
rs17677991
rs762679
rs10905255
rs1332941
rs4724
rs932002
SNP
-0.06
-0.04
-0.02
0.00
0.02
Effect size (b)
